# Distinct virulence ranges for infection of mice by *Bordetella pertussis* revealed by engineering of the sensor-kinase BvgS

**DOI:** 10.1371/journal.pone.0204861

**Published:** 2018-10-11

**Authors:** Elodie Lesne, Loic Coutte, Luis Solans, Stephanie Slupek, Anne-Sophie Debrie, Véronique Dhennin, Philippe Froguel, David Hot, Camille Locht, Rudy Antoine, Françoise Jacob-Dubuisson

**Affiliations:** 1 Univ. Lille, Lille, France; 2 CNRS UMR 8204, Lille, France; 3 Inserm U1019, Lille, France; 4 CHU Lille, Lille, France; 5 Institut Pasteur de Lille, Centre d’Infection et d’Immunité de Lille, Lille, France; 6 CNRS UMR 8199, European Genomic Institute for Diabetes, Lille, France; Instituto Butantan, BRAZIL

## Abstract

The whooping cough agent *Bordetella pertussis* coordinately regulates the expression of its virulence factors with the two-component system BvgAS. In laboratory conditions, specific chemical modulators are used to trigger phenotypic modulation of *B*. *pertussis* from its default virulent Bvg^+^ phase to avirulent Bvg^-^ or intermediate Bvg^i^ phases, in which no virulence factors or only a subset of them are produced, respectively. Whether phenotypic modulation occurs in the host remains unknown. In this work, recombinant *B*. *pertussis* strains harboring BvgS variants were tested in a mouse model of infection and analyzed using transcriptomic approaches. Recombinant BP-Bvg_Δ65,_ which is in the Bvg^i^ phase by default and can be up-modulated to the Bvg^+^ phase *in vitro*, could colonize the mouse nose but was rapidly cleared from the lungs, while Bvg^+^-phase strains colonized both organs for up to four weeks. These results indicated that phenotypic modulation, which might have restored the full virulence capability of BP-Bvg_Δ65,_ does not occur in mice or is temporally or spatially restricted and has no effect in those conditions. Transcriptomic analyses of this and other recombinant Bvg^i^ and Bvg^+^-phase strains revealed that two distinct ranges of virulence gene expression allow colonization of the mouse nose and lungs, respectively. We also showed that a recombinant strain expressing moderately lower levels of the virulence genes than its wild type parent was as efficient at colonizing both organs. Altogether, genetic modifications of BvgS generate a range of phenotypic phases, which are useful tools to decipher host-pathogen interactions.

## Introduction

*Bordetella pertussis* is the agent of an acute respiratory disease, whooping cough. Despite the current global vaccination coverage of approximately 86% of the population, this bacterium still causes 16 million cases and 200,000 deaths per year [1]. To colonize the human respiratory tract, *B*. *pertussis* produces a number of virulence factors, notably adhesins and toxins, whose expression is regulated by a two-component system called BvgAS [2]. BvgS is a sensor-kinase protein that auto-phosphorylates and transfers the phosphoryl group via a complex cascade of phosphorylation to BvgA, the response regulator. BvgA acts as a canonical transcriptional activator in its phosphorylated form [3].

The virulence status of *B*. *pertussis* is defined by three phases during which specific genes are expressed, a phenomenon referred to as phenotypic modulation [4]. At 37°C, in standard culture conditions, *B*. *pertussis* expresses all its virulence-activated genes (‘*vags*’), which defines the Bvg^+^ phase. In laboratory conditions, in presence of so-called negative modulators such as nicotinate or MgSO_4_ at millimolar concentrations, or in conditions of nutrient restriction, the bacteria shift to the avirulent Bvg^-^ phase [5]. During this phase, the so-called virulence-repressed genes (‘*vrgs*’) are up-regulated, while the *vags* are silent. An intermediate phase (Bvg^i^) was described at intermediate concentrations of modulators, in which a subset of *vags*, including *bvgAS* and genes coding for adhesins, are expressed [6]. Those were defined as ‘early’ *vags*, as they are the first genes of the Bvg regulon to be up-regulated following a shift from modulating to non-modulating conditions [7, 8]. In addition to the early *vags*, at least one gene specific of the Bvg^i^ phase, *bipA*, is overexpressed in the Bvg^i^ phase [9, 10]. Phenotypic modulation depends on the concentration of phosphorylated BvgA, itself related to the enzymatic activity of BvgS [4]. In basal conditions, BvgS functions as a kinase, while in modulated conditions it shifts to phosphatase activity [3, 11–13]. The signals that may cause the phase shift from Bvg^+^ to Bvg^i^ or Bvg^-^ in the host respiratory tract remain unknown.

BvgS is a large, dimeric protein that serves as a prototype for a family of poorly characterized Venus flytrap domains (VFT)-containing sensor-kinases [12, 14]. In the homodimer, each monomer is composed of two periplasmic VFT domains possibly involved in signal perception, followed by a transmembrane segment, a cytoplasmic Per-ArnT-Sim (PAS) domain, a histidine-kinase domain and two other domains, the receiver and the phosphotransfer domains, involved in a phosphorelay to BvgA. We have shown that the VFT1 domains of BvgS open and close, like typical VFT proteins, while the VFT2 domains are permanently closed [12]. Rather puzzlingly, however, negative chemical modulators such as nicotinate bind to the latter domain [13, 15]. Artificially closing the VFT1 domains strongly reduces BvgS kinase activity [12]. As VFT domains generally close upon binding their specific ligands [16, 17], this observation suggests that the VFT1 domains might perceive chemical signals that down-modulate BvgS activity at some stage of the infection.

In BvgS and the majority of its homologs, the transmembrane domain is followed by a two-helix linker called linker 1 that leads to the PAS domain. This is followed by a second two-helix linker, linker 2, that leads to the α-helical Dimerization and Histidine phosphorylation moiety (DHp) of the kinase domain [18, 19]. However, a sizeable proportion of BvgS homologs are devoid of a PAS domain and flanking linkers. Instead, a two-helix linker called the linker X directly connects the transmembrane segment to the DHp domain [20]. The two-helix linkers form coiled coils that regulate the BvgS enzymatic activity [18, 19]. We have built several chimeras by replacing the region between the TM and DHp domains of BvgS with those from homologs devoid of PAS domain [18, 20]. Among the various regulation phenotypes of those chimeras, that of the so-called BvgS_Δ65_ is inverted relative to that of BvgS [20]. Thus, BvgS_Δ65_ shows low kinase activity at the basal state, most likely corresponding to the Bvg^i^ phase, while chemical modulation or closure of the VFT1 domains triggers a large increase of kinase activity, shifting the bacteria to the Bvg^+^ phase [20]. In this study, we characterized this and other BvgS chimeras using transcriptomic analyses and a mouse model of infection. We obtained no evidence for a shift of BvgS_Δ65_ to the kinase mode of activity in the mouse during the time course of infection. In addition, we identified different ranges of virulence factor expression for colonization of, and persistence in, distinct sites of the mouse respiratory tract.

## Materials and methods

### Strains and plasmids

The strains used to perform animal experiments, BPSM, BPSM_SS1_ (previously called BPSM_BvgS-E113C+N177C_), BP-BvgS_Δ65_ and BP-BvgS_Δ65-SS1_ (previously called BvgS_Δ65-VFT1-SS_) were described previously [12, 20]. In all of them, expression of the *bvgS* variants from the natural chromosomal locus was achieved by allelic exchange as described in [12]. Similarly, to construct BP-BvgS_T733M_ the mutation of interest was introduced by mutagenesis on a pUC19 derivative containing the appropriate region of the *bvgS* gene, followed by cassette exchange in pUC19mint, transfer of the EcoRI-HindIII fragment into pSORTP1 and homologous recombination into BvgS_newΔAS_ as in [12].

The BvgS_Δ65 R572L_ variant was expressed from a plasmid for activity measurement. It was constructed by mutagenesis on a pUC19 derivative containing the region of interest, followed by cassette exchange in pUC19mpla and then in pBBRmpla [12]. The recombinant *B*. *pertussis* strains were obtained by introducing the pBBRmpla variant by conjugation in *B*. *pertussis* BPSM_newΔAS_ carrying the chromosomal *ptx-lacZ* transcriptional fusion [12]. *bvgS*_*Δ65*_ was cloned on a plasmid in a similar manner for comparison.

### Animal experiments

After 36 h of growth on standard Bordet-Gengou (BG)-blood plates or plates containing 50 mM MgSO_4_ to modulate the various strains, bacteria were resuspended in sterile PBS to 10^6^ bacteria per 20 μL. After intraperitoneal anesthesia with a mixture of ketamine, atropine and valium, female 6-weeks-old JAX BALB/cByJ mice from Charles River were infected by intranasal inoculation with 10^6^ bacteria. Groups of 5 animals per bacterial strain were sacrificed by cervical dislocation after 3 h and 3, 7, 14 or 28 days post-inoculation in the first experiment, and groups of 3 animals after 3 h and 7, 14 or 21 days in the second. Noses and lungs were collected and homogenized using an Ultra Turrax apparatus. Serial dilutions were performed in PBS and plated on BG agar plates to count the bacteria. All the experiments were carried out in accordance with the guidelines of the French Ministry of Research regarding animal experiments, and the protocols were approved by the Ethical Committees of the Region Nord Pas de Calais and the Ministry of Research (agreement number APAFIS#9107–201603311654342 V3).

### RNA extractions

*B*. *pertussis* strains were grown on BG agar plates for 2 days at 37°C and then cultured in modified Stainer Scholte (SS) liquid medium supplemented when indicated with 50 mM MgSO_4_ at 37°C under agitation. The bacterial cultures were stopped at mid exponential phase (OD_600_ = 2) by adding 1 mL of a mixture of 5:95 phenol:ethanol (v:v) to 4 mL of bacterial suspensions. Bacteria were pelleted, and total RNA was extracted using TriReagent (Invitrogen) following the manufacturer’s instructions. Genomic DNA was removed by DNase I treatment (Sigma Aldrich).

### Illumina RNA sequencing

RNA-seq experiments were performed with two independent cultures of BPSM and BPSM grown in the presence of 50 mM MgSO_4_, and with single cultures of BPSM_SS1_, BP-BvgS_Δ65_, BP-BvgS_Δ65-SS1_ and BP-BvgS_T733M_. For each RNA-seq sample, DNA-depleted total RNA was treated with the Ribo-Zero rRNA Removal Kit (Illumina) following the manufacturer’s recommendations. The rRNA-depleted RNA was then used to make the Illumina library using the TruSeq RNA Library Preparation Kit, following sequencing on an Illumina NextSeq 500 benchtop sequencer on SR150 high output run mode. The RNA-seq data of each sample were analyzed using Rockhopper v2.0.3 with the default parameters to calculate the RPKM value for each CDS using the *Bordetella pertussis* Tohama I BX470248 genome annotation [21]. The RNA-seq data reported in this paper have been deposited in the Sequence Read Archive, www.ncbi.nlm.nih.gov/sra (submission SUB4097406; NCBI BioProj PRJNA474836; BioSample accessions: SAMN09374659, SAMN09374660, SAMN09374661, SAMN09374662, SAMN09374663, SAMN09374664, SAMN09374665, SAMN09374666).

### Generation of cDNA and quantitative real-time polymerase chain reaction (qRT-PCR)

Fifteen micrograms of total RNA were treated with DNAse I, and then 500 ng of total RNA was reverse-transcribed using the Verso cDNA synthesis kit (Thermo Scientific). Polymerase chain reaction (PCR) was performed on 30 ng of cDNA using a LightCycler 480 SYBR Green I Master kit (Roche) and a Roche LightCycler 480 Instrument II. The efficiency for each primer pair was determined by serial dilutions. The experiments were performed three times for BPSM, MgSO_4_-treated BPSM, BP-BvgS_Δ65_, MgSO_4_-treated BP-BvgS_Δ65,_ BPSM_SS1_, BPSM_Δ65 SS1_, BPSM_T733M_ and BP-BvgS_Δ65-_rev 79_,_ twice for BP-BvgS_Δ65-_rev 80 and from single biological sampling for the other BP-BvgS_Δ65-_rev strains. For each sample, at least three technical replicates were performed. The results were analyzed with the Light Cycler 480 software. The expression of the housekeeping gene *bp3416* was used as reference to normalize the expression of the genes of interest.

### Extraction of genomic DNA and sequence determination

The various segments of the *bvgS* gene were amplified from clarified lysates obtained by heating bacteria resuspended in H_2_O at 95°C for 30 min, using the pairs of primers described in [22], and the amplicons were sequenced by the Sanger method. DNAseq experiments were performed on genomic DNA extracted using the Genomic tip 100/G kit (Qiagen). The sequencing libraries were prepared with the Nextera XT sample prep kit (Illumina) following the manufacturer’s instruction and sequenced on HiSeq 2500 with 2x250 bp reads. The read mapping was performed with CLC Genomics Workbench V11, and variants were searched using the Basic Variant Detection module.

### β-galactosidase assay

β-galactosidase assays were performed as described previously [12] with 3 different clones at different times, and the means and standard errors of the means were determined. TCEP was added to the cultures for 6 or 16 hours prior to harvesting the bacteria.

### Statistical analyses

Statistical analyses were performed using an unpaired t test for β-galactosidase results, and using the one-way ANOVA followed by Bonferroni’s multiple comparison post-test for the animal experiments and qRT-PCR results. For the RNA-seq data, the adjusted p-values were determined using the Benjamini-Hochberg procedure in Rockhopper.

## Results

### Enhancement of animal colonization by reversion of the BvgS_Δ65_ regulation phenotype

**Fig 1:**
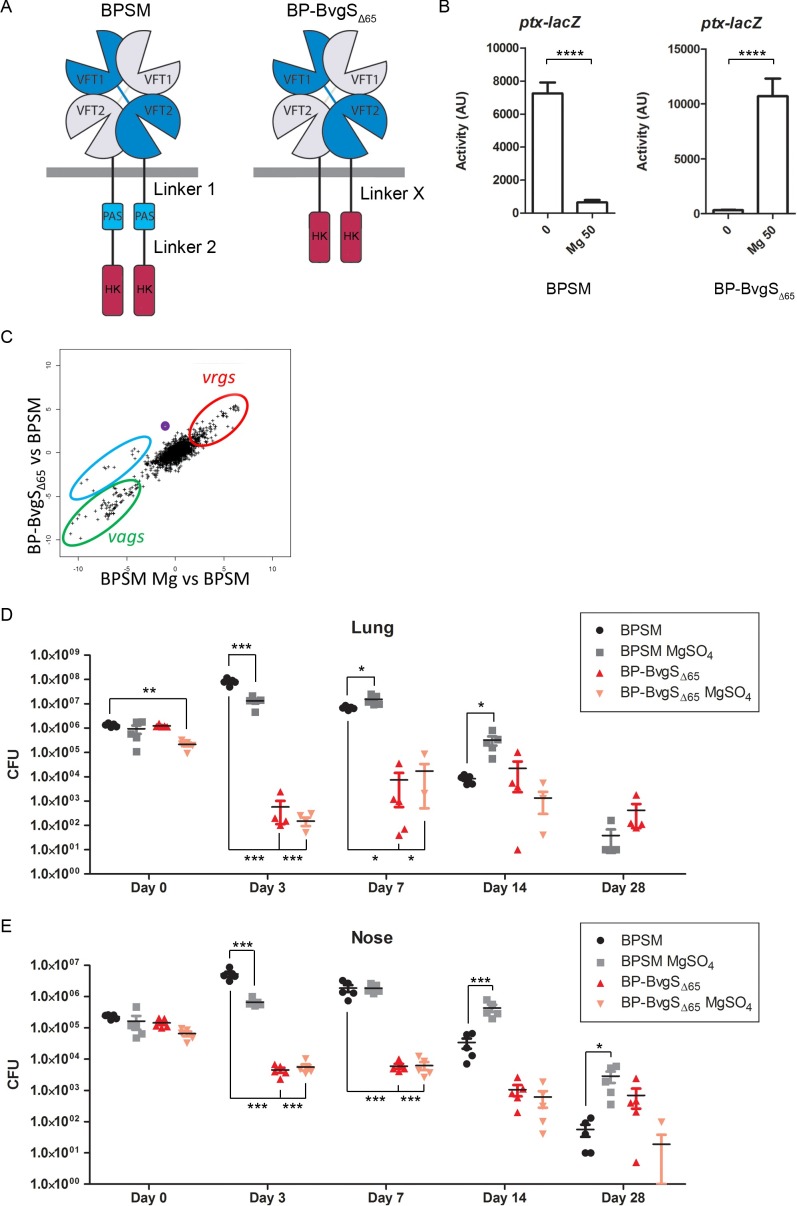
Characterization of BP-BvgS_Δ65_ and its colonization of the mouse respiratory tract by comparison with control strain BPSM. A. Schematic representation of wild type BvgS as found in the control strain BPSM and of the BvgS_Δ65_ variant_._ The VFT domains and Histidine Kinase (HK) domains are connected to one another with the two-helix linkers 1 and 2 and the intervening PAS domain in BPSM, or with a single two-helix linker X in BPSM-BvgS_Δ65_. The receiver and Hpt domains of BvgS were omitted for clarity. B. Activities of the BvgS variants as measured using the *ptx-lacZ* reporter system. The strains were grown in standard conditions (0) or with 50 mM MgSO_4_ (Mg 50). The measurements were performed at least three times, and the means and standard errors of the mean are given. Statistical analyses were performed, and significant p values are indicated (****, p<0.0001). C. Illumina RNA sequencing was performed for BPSM, BPSM grown in the presence of 50 mM MgSO_4_ (BPSM Mg), and BP-BvgS_Δ65_. The trancriptomes of BPSM grown in standard conditions and in 50 mM MgSO_4_ were used as the reference Bvg^+^ and Bvg^-^ transcriptomes, respectively. The data are plotted as the ratios of gene expression in BP-BvgS_Δ65_ relative to BPSM (y-axis) versus the ratios of gene expression in modulated BPSM (denoted BPSM Mg) relative to BPSM (x-axis). Thus, genes found on a straight line starting from the origin with a slope of 1 are regulated similarly in BP-BvgS_Δ65_ and in modulated BPSM, while genes found above this line are expressed at higher levels in the former than in the latter. Two distinct groups of *vags* are circled in green and blue, with the blue oval encompassing some early *vags*. Most *vrgs* are circled in red, and *bipA* is shown in purple. The complete datasets of these experiments are presented in S1 Table. D and E. Numbers of colony-forming units (CFUs) recovered at the indicated time points from the lungs (D) or the noses (E) of mice infected with BPSM or BP-BvgS_Δ65_. Prior to inoculation, the bacteria were grown in standard or modulating conditions (addition of 50 mM MgSO_4_). Five mice were sacrificed at each time point for each bacterial strain. The means and standard errors of the means are shown. Statistical analyses were performed for each data point using the corresponding BPSM data point as a control. Only significant p values are indicated (***, p<0.001; **, p<0.01; *, p<0.05).

The BP-BvgS_Δ65_ recombinant strain shows an inverted regulation phenotype relative to its wild type (wt) parent BPSM in laboratory conditions [20], as chemical modulation shifts BvgS _Δ65_ to a high-kinase mode of activity (Fig 1A and 1B). We characterized the full gene expression profile of BP-BvgS_Δ65_ in the ‘default state’ (ie, not modulated) by performing Illumina RNA sequencing (RNA-seq) experiments and compared it with that of BPSM modulated or not (Fig 1C; S1 Table). The transcription profile of BPSM was in good agreement with previous reports [23–25], with high transcription levels of the *vags* and low transcription levels of the *vrgs* (Fig 1C, green and red ovals; S1 Table). Except for a few sets of genes, the gene expression profile of BP-BvgS_Δ65_ was rather close to that of modulated BPSM. Thus, expression of late *vags*, *e*.*g*. coding for PTX or the type III secretion system, decreased to the same levels as in modulated BPSM (Fig 1C, green oval; S1 Table). In contrast and congruent with the Bvg^i^ phase, the expression levels of early *vags* such as *fhaB*, *fimBCD* or *fhaC* in BP-BvgS_Δ65_, were similar to those in BPSM (Fig 1A, blue oval), and *bipA* (*bp1112*), the hallmark gene of the Bvg^i^ phase in *B*. *pertussis*, was expressed at higher level in BP-BvgS_Δ65_ than in BPSM grown in standard or modulated conditions (Fig 1C, purple circle) [23]. Additionally, some other *vags*, like *fhaS* (*bp2667*), *fmtB* (*bp2936*), *prn* (*bp1054*), and other genes including *lgmABC* (*bp0397*, *bp0398* and *bp0399*) and *putA* (*bp2749*) were expressed at higher levels in BP-BvgS_Δ65_ than in modulated BPSM (S1 Table). Most of the *vrgs* were expressed at levels similar to those in modulated BPSM, which was not previously reported for the Bvg^i^ phase (Fig 1A, red ovals) [23]. Altogether, those results indicate that the default state of BP-BvgS_Δ65_ is the Bvg^i^ phase.

As *in vitro* modulation shifts BP-BvgS_Δ65_ to the Bvg^+^ phase, we investigated whether animal experiments might reveal specific conditions present in the mammalian respiratory tract that induce phenotypic modulation, thus causing a shift of BP-BvgS_Δ65_ to the colonization-proficient, Bvg^+^ phase. If this occurred, the ‘up-modulated’ recombinant bacteria should be able to colonize the animal lungs, while they would be cleared quickly if they remained in the Bvg^i^ phase, as described earlier using other Bvg^i^-phase strains [26, 27]. In contrast, we did not expect them to be rapidly cleared from the mouse noses, as *B*. *pertussis* locked in the Bvg^i^ phase was reported to survive in the mouse upper respiratory tract in a way similar to virulent bacteria [26, 27]. Mouse colonization experiments were thus performed with BP-BvgS_Δ65_ cultured in standard conditions (i.e., 37°C without modulator) prior to inoculation, or at 37°C in the presence of 50 mM MgSO_4_ to set the bacteria in the Bvg^+^ phase prior to colonization. We reasoned that if the bacteria encountered modulating conditions at a late stage of mouse colonization rather than early on, pre-modulation of BP-BvgS_Δ65_ might facilitate initial survival in the animals, before induction of virulence factor expression resulting from *in vivo* modulation. If no modulation occurred *in vivo*, we expected pre-modulated BP-BvgS_Δ65_ to progressively lose virulence factor expression and therefore to be cleared like its non-modulated counterpart. The wt control strain, BPSM, was cultured in the same two conditions before mouse inoculation. The mice were infected intranasally with 10^6^ bacteria. After 3 h, approximately 10^6^ and 10^5^ bacteria were found in the lungs and in the noses of mice infected with the BPSM control or with BP-BvgS_Δ65_ (Fig 1D and 1E). The colonization profiles by BPSM were as typically reported. At day 3, the bacterial loads increased in both organs, the bacteria were found in similar numbers at day 7, and then their numbers markedly decreased at days 14 and 28. Bacterial multiplication and clearance from both organs appeared to be slightly delayed for BPSM chemically down-modulated prior to inoculation, possibly because virulence factor production was initiated only when the bacteria encountered the *in vivo* environment of the animals’ respiratory tract and shifted to the Bvg^+^ phase.

For BP-BvgS_Δ65_, in contrast, no bacterial multiplication was observed at day 3 in either organ. On the contrary, marked decreases of the bacterial loads were seen irrespective of prior up-modulation of the bacteria. Thus, *in vitro* modulation before inoculation failed to restore a wt-like colonization profile. The fast decreases of the BP-BvgS_Δ65_ populations in both organs do not support the hypothesis that up-modulating signals were present in the respiratory tract that might have shifted BvgS_Δ65_ to a high-kinase mode of activity.

**Fig 2:**
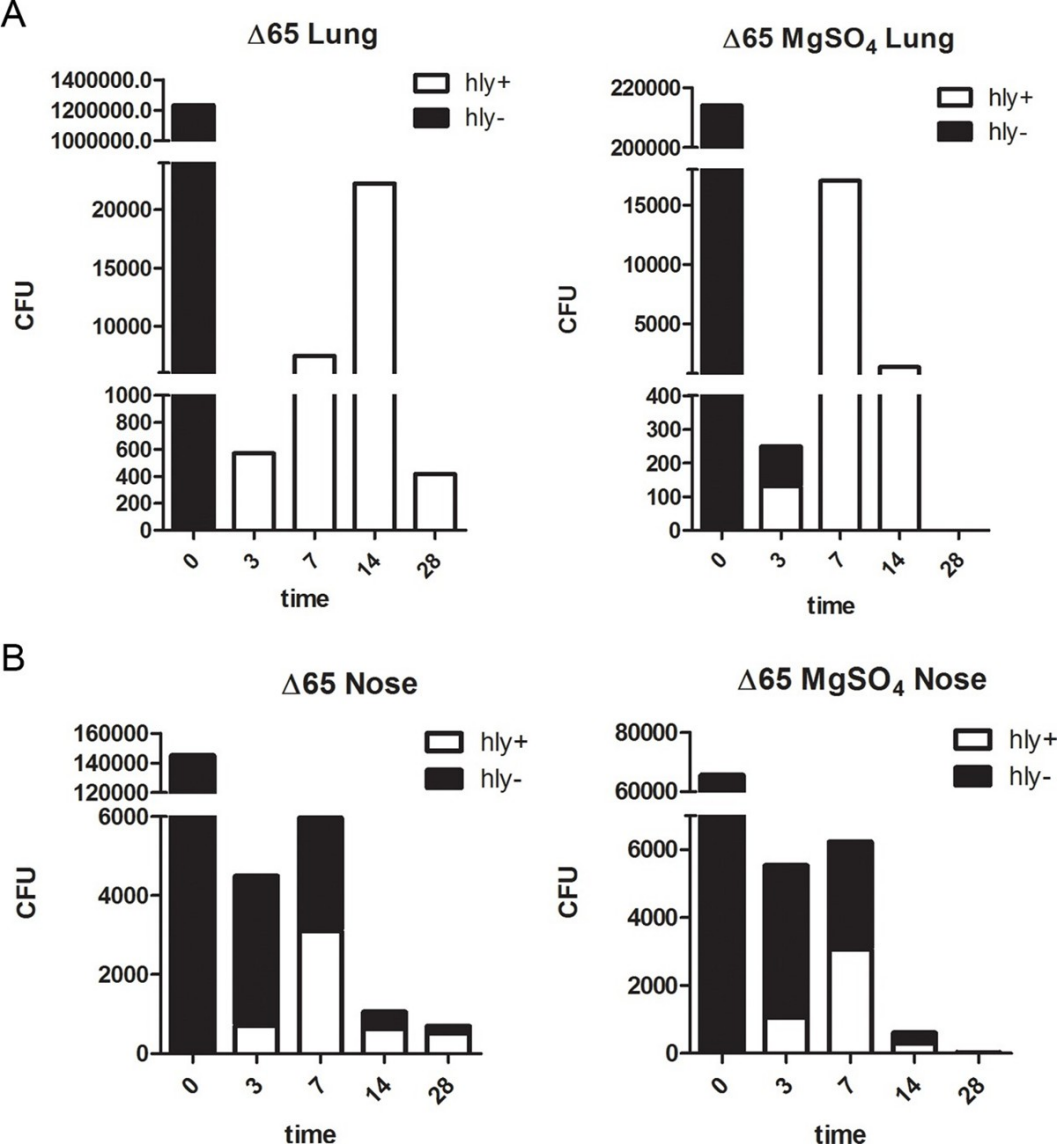
Time course of the appearance of hemolytic colonies in the noses and lungs of mice infected with BP-BvgS_Δ65_. A and B. Aggregate numbers of hemolytic colonies (hly+) in the samples recovered from the lungs (A) and the noses (B), at the indicated time points in days post-inoculation.

At later time points, however, BP-BvgS_Δ65_ appeared to survive at similar levels to BPSM. BP-BvgS_Δ65_ is non-hemolytic on blood agar plates in non-modulated culture conditions, due to the lack of or the very low-level expression of the hemolysin/adenylate cyclase gene *cyaA*, a late *vag* whose transcription requires high concentrations of phosphorylated BvgA [8]. Intriguingly, however, all colonies recovered at day 3 from the lungs of mice infected with non-modulated BP-BvgS_Δ65_, and 40% of those recovered from the lungs of mice infected with BP-BvgS_Δ65_ modulated prior to infection were hemolytic (Fig 2A). At day 7, only hemolytic colonies were recovered from the lungs of the two groups of mice. Hemolytic colonies were also recovered from the noses, in low proportions at day 3 that appeared to increase at day 7 (Fig 2B). Nevertheless, non-hemolytic clones were detected in the noses up to day 28. The hemolytic phenotype was maintained after re-streaking those clones on Bordet Gengou agar-blood plates with or without modulators, suggesting that the bacteria were locked in the Bvg^+^ phase.

The hemolytic bacteria appear to have undergone a reversion from the Bvg^i^ phase to the Bvg^+^ phase in most of the animals. Possibly because of this reversion, we observed a moderate increase of the bacterial loads of BP-BvgS_Δ65_ in the lungs between days 3 and 7, for both the bacteria chemically modulated and those not modulated prior to inoculation (Fig 1D and 1E). Nevertheless, the bacterial loads remained 1000-fold lower than those of BPSM even at their peak at day 7. The numbers of bacteria in the lungs of those two groups of mice leveled off at day 14 and decreased thereafter. In the noses, where the proportions of hemolytic colonies were initially lower, the bacterial counts remained steady between days 3 and 7 and started to decrease from day 14. Thus, infection of mice with BP-BvgS_Δ65_ resulted in the appearance of hemolytic variants whose proportions in the bacterial populations recovered from both organs increased over time. This suggested that it could be due to selective pressure in mice. As only 0.1% of the initial bacterial loads were recovered from the lungs after 3 days, the remaining 99.9% bacteria, which were presumably non-revertants, most likely died in the first three days. The potential selective pressure for virulent bacteria—using the hemolytic phenotype as a proxy for virulence- appears to be less intense in the nose than in the lungs, as more than 40% of the BP-BvgS_Δ65_ bacteria recovered from the noses were non-hemolytic at day 14 (Fig 2B).

### Colonization profiles by distinct Bvg^i^-phase bacteria

Since hemolytic revertants were obtained from several animals, the most likely explanation is that they spontaneously arose at low frequency in our master stock of BP-BvgS_Δ65_ and were selected for by the host environment. However, when BP-BvgS_Δ65_ was streaked on blood-agar plates and incubated for 5 days at 37°C, no hemolytic colony was detected, indicating that hemolytic bacteria, if present, would represent a very small proportion of the stock. Furthermore, deep Illumina whole-genome sequencing of BP-BvgS_Δ65_ did not reveal any mutations.

**Fig 3:**
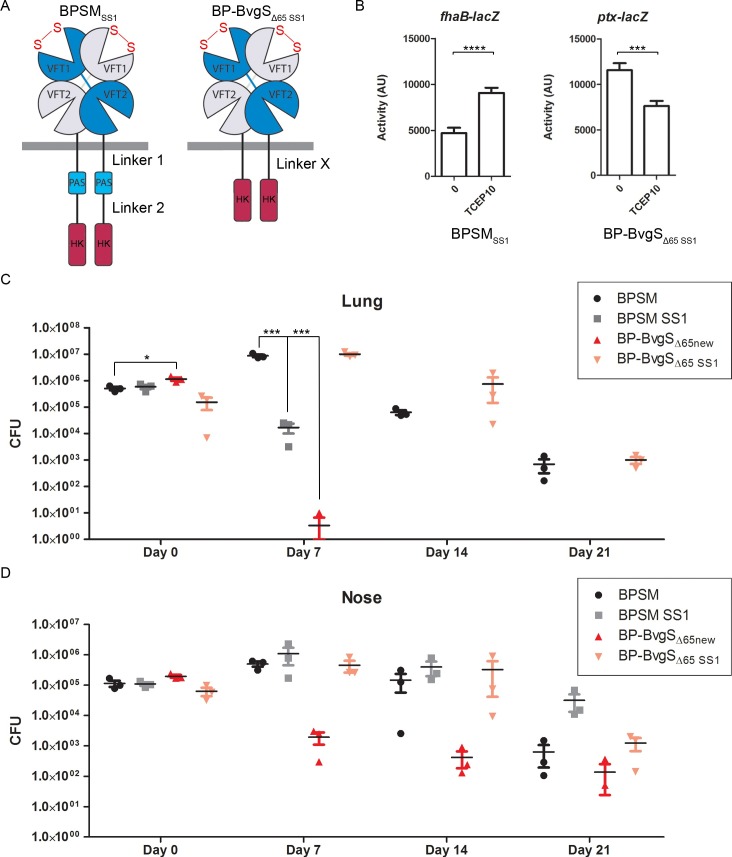
Characterization of strains used in this study and colonization of the lungs and noses of mice. A. Schematic representation of the BvgS variants present in BPSM_SS1_ and BPSM-BvgS_Δ65-SS1_ (see legend of Fig 1). Disulfide bound formation between the two lobes of the VFT1 domain is indicated with ‘S-S’. B. Activities of the variants as measured using the *ptx-lacZ* or *fhaB-lacZ* reporter systems. The strains were grown in standard conditions (0), and where indicated 10 mM Tris(2-carboxyethyl)phosphine hydrochloride (TCEP 10) was added to reduce the S-S bond in VFT1. The measurements were performed at least three times, and the means and standard errors of the mean are given. Statistical analyses were performed, and significant p values are indicated (****, p<0.0001, *** p<0.001). C and D. Numbers of colony-forming units (CFUs) recovered at the indicated time points from the lungs (C) or the noses (D) of mice infected with BPSM or BP-BvgS_Δ65_. Prior to inoculation, the bacteria were grown in standard conditions. Three mice were sacrificed at each time point for each bacterial strain. The means and standard errors of the means are shown. Statistical analyses were performed for each data point using the corresponding BPSM data point as a control. Significant p values are indicated (***, p<0.001; *, p<0.05). The other data present p-values >0.05.

We nevertheless performed a new round of single colony isolation starting from our initial BP-BvgS_Δ65_ stock, which yielded BP-BvgS_Δ65new_, and we performed another mouse colonization experiment with the newly isolated clone. In this second experiment, in addition to BPSM and BP-BvgS_Δ65new_ we included two other strains that express BvgS variants with different levels of enzymatic activities, BPSM_SS1_ [12] and BP-BvgS_Δ65 SS1_ [20] (Fig 3A and 3B). In BPSM_SS1_, two selected residues at the lips of the lobes of the VFT1 domain of BvgS were replaced with Cys residues, which results in the formation of an inter-lobe disulfide (S-S) bond that artificially closes that domain when the bacteria are growing in liquid culture [12]. This forced closure abolished BvgS kinase activity in both standard and modulated conditions using the *ptx-lacZ* reporter [12]. However, using the *fhaB-lacZ* reporter in standard growth conditions, we observed in this study that BvgS_SS1_ expresses the *fhaB* gene, an early vag whose transcription requires only small concentrations of phosphorylated BvgA [7, 8] (Fig 3B). The addition of a reducing agent, TCEP, to the bacterial cultures increased the β-gal activity with the same reporter. Thus, in non-modulating conditions, BvgS_SS1_ appears to be in a ‘low-kinase’ (Bvg^i^) mode of activity, rather than in the *bona fide* phosphatase mode. Conversely, introduction of the same S-S bond in BvgS_Δ65_, yielding BvgS_Δ65-SS1_, shifted it to a high-kinase mode of activity, as shown with the *ptx-lacZ* reporter in standard conditions [20] (Fig 3B). Upon addition of TCEP to the culture, the β-gal activity decreased significantly but not fully, suggesting that S-S bond reduction was most likely incomplete in those conditions.

In the second animal experiment, two Bvg^+^-phase strains, BPSM and BP-BvgS_Δ65-SS1_, and two Bvg^i^-phase strains, BP-BvgS_Δ65new_ and BPSM_SS1_, were thus used to inoculate mice. The bacteria were counted in their lungs and noses over the time course of infection. The colonization profiles of both organs by BP-BvgS_Δ65-SS1_ were similar to those of BPSM (Fig 3C and 3D). This indicates that formation of the S-S bond in the VFT1 domains of BvgS_Δ65 SS1_ enabled the bacteria to express the virulence factors necessary for colonization and survival in both organs. In contrast, the two Bvg^i^-phase strains, BP-BvgS_Δ65new_ and BPSM_SS1_, were rapidly cleared from the lungs but colonized the noses of mice.

In contrast to the first animal experiment, no hemolytic colonies of BP-BvgS_Δ65new_ were obtained from either organ at any time point. This supported the hypothesis that the initial BP-BvgS_Δ65_ bacterial stock used for the first experiment contained a very small proportion of revertants that were not detected on blood-agar plates prior to inoculation of the mice. In the lungs, BP-BvgS_Δ65new_ was rapidly eliminated and was not detectable after day 7. In the noses, this strain did not multiply, and the bacterial load markedly decreased at day 7, followed by a milder decrease thereafter.

In the lungs, BPSM_SS1_ did not multiply and was rapidly cleared, though not quite as quickly as BP-BvgS_Δ65new_. In contrast, BPSM_SS1_ persisted in the noses in a manner similar to BPSM, or even better at day 21. Thus, whereas the recombinant strains that express BvgS variants displaying intermediate modes of activity were rapidly cleared from the lungs, they survived much longer in the noses. Intriguingly, BPSM_SS1_ persisted in the noses at higher bacterial counts than BP-BvgS_Δ65new_. The observation that the nose colonization profiles of two strains in the Bvg^i^ phase were not identical indicates that they might display slightly different gene expression patterns.

#### Expression range of the various recombinant strains

**Fig 4:**
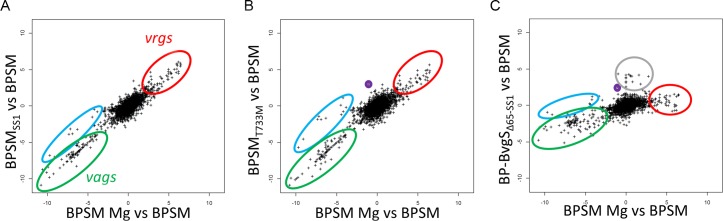
Transcriptomic analyses of the recombinant strains. Illumina RNA sequencing was performed for BPSM_SS1_ (A), BPSM_T733M_ (B) and BP-BvgS_Δ65-SS1_ (C). The transcriptomes of BPSM grown in standard conditions or in 50 mM MgSO_4_ were used as the reference Bvg^+^ and Bvg^-^ transcriptomes. The data are plotted as in Fig 1C. Two distinct groups of *vags* are circled in green and blue, with the blue circle encompassing some early *vags*. Most *vrgs* are circled in red, and genes coding for the chemotaxis and flagellar operons are circled in grey in panel C, as they stand out in that particular strain. *bipA* is shown in purple. The complete datasets of those experiments are presented in S1 Table.

We performed RNA-seq experiments on BPSM_SS1_ and BP-BvgS_Δ65-SS1_ to characterize their gene expression profiles (Fig 4A and 4C). An additional strain, BPSM_T733M_, which represents a prototypical Bvg^i^-phase-locked strain, was included in the experiments (Fig 4B). The T^733^M mutation, which localizes to the DHp domain of BvgS, spontaneously occurred in *Bordetella bronchiseptica* and led to the initial description of the Bvg^i^ phase [9]. This mutation was introduced in the BPSM chromosome by allelic exchange to serve as the Bvg^i^ reference strain for RNA-seq.

The gene expression profiles of BPSM_SS1_ and BPSM_T733M_ were broadly similar to each other and rather close to that of BP-BvgS_Δ65_ (compare Fig 4A and 4B with Fig 1C; S1 Table). Subtle differences were nevertheless found among the Bvg^i^-phase strains, notably regarding the levels of expression of specific *vags* (Fig 1C and Fig 4A–4C, blue ovals; S1 Table). Furthermore, *bipA* (*bp1112*), a specific marker of the Bvg^i^ phase [23], was overexpressed in both BP-BvgS_Δ65_ and BPSM_T733M_ (purple circles), but not to the same extent in BPSM_SS1_. Altogether, BP-BvgS_Δ65,_ BPSM_T733M_ and BPSM_SS1_ present transcriptomic patterns characteristic of the Bvg^i^ phase, but within a certain range of gene expression levels. The reason why BPSM_SS1_ was more efficient at colonizing the mice noses than BP-BvgS_Δ65_ is difficult to ascribe to specific differences between their transcriptomes.

BP-BvgS_Δ65-SS1_ expressed several *vags*, including *ptx*, *vag8* (*bp2315)*, *tcfA* (*bp1201*), *brkA* (*bp3494*) and *bfrD* (*bp856*), at significantly lower levels than BPSM, but higher than the three Bvg^i^-phase strains (S1 Table). The expression of *bipA* in BP-BvgS_Δ65-SS1_ was higher than in BPSM and in the same range as in the Bvg^i^-phase strains. BP-BvgS_Δ65-SS1_ also expressed the genes of the chemotaxis and flagellar operons at higher levels than modulated BPSM (Fig 4C, grey circle). BP-BvgS_Δ65-SS1_ is thus closer to the Bvg^+^ phase than the other variants but with lower expression levels of several *vags* than BPSM, although it colonized mice as efficiently as BPSM. Genetic engineering of BvgS can thus generate a range of intermediate phenotypes.

**Fig 5:**
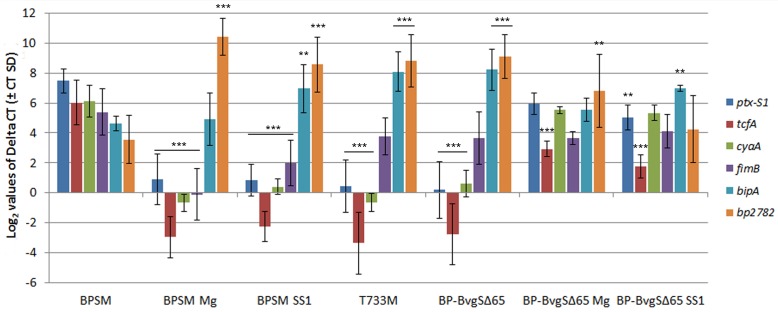
Quantitative RT-PCR analysis of selected genes in the recombinant strains. The values represent the Log2 values of the mean expression of each gene normalized to *bp3416* using the 2ΔCt method. The error bars represent the Ct standard deviations. Modulation with 50 mM MgSO_4_ (denoted Mg) was performed on BPSM and BP-BvgS_Δ65_. The results represent biological triplicates. In all cases, the measurements were performed in triplicates. Statistical analyses were performed using gene expression in BPSM as a control and indicated as follows: ***, p<0.001, **, p<0.01, *, p<0.05.

We also performed quantitative real-time polymerase chain reaction (qRT-PCR) experiments to quantify the transcripts of selected genes in the various strains (Fig 5). We included late *vags* (*cya*, *ptx-S1*, *tcfA*), an early *vag* (*bp1881*, *i*.*e*. *fimB*), the intermediate phase marker *bipA*, and the *vrg bp2782*. We tested the effect of modulation on gene expression in BP-BvgS_Δ65_.

The qRT-PCR data showed that the strains expressing BvgS_Δ65_, BvgS_T733M_ and BvgS_SS1_ have similar patterns of expression that represent the Bvg^i^ phase. This analysis also confirmed that BP-BvgS _Δ65_ treated with MgSO_4_ and BP-BvgS_Δ65 SS1_ are closer to the Bvg^+^ phase than the three Bvg^i^-phase strains, although they expressed some *vags* at lower levels than BPSM. Thus, the BvgS variants analyzed in this work display a range of gene expression patterns between the fully Bvg^+^ and Bvg^-^ phases, which most likely correlate with their respective behaviors in animal colonization.

### Identification of a mutation in hemolytic colonies of BP-BvgS_Δ65_

**Fig 6:**
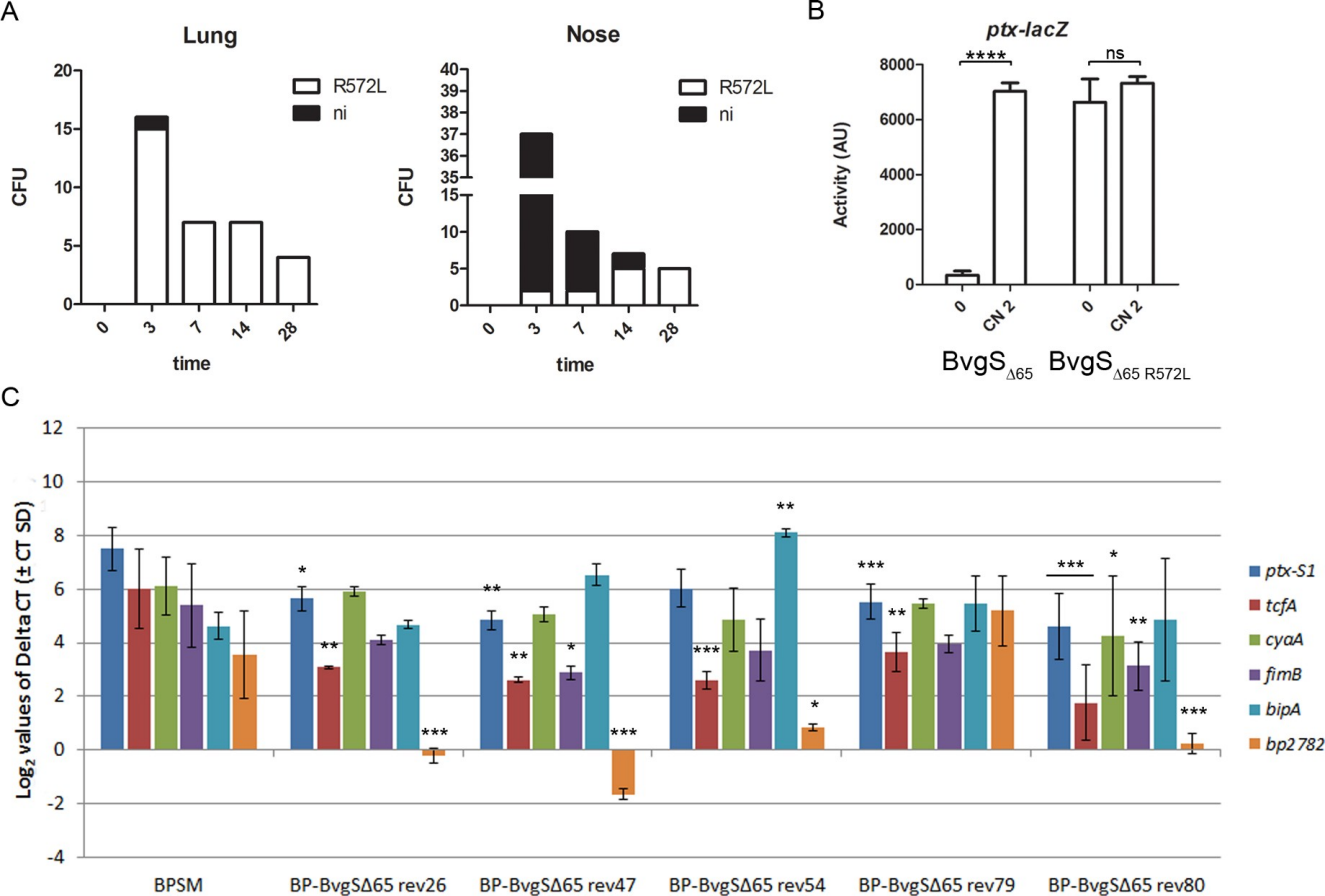
Characterization of hemolytic variants of BP- BvgS_Δ65_. A. Numbers of hemolytic colonies selected for sequencing that present the R^572^L mutation. The variants were isolated from the lungs (left panel) and noses (right panel) of the mice. The cause of the hemolytic phenotype of the clones that do not carry the above mutation is not identified (ni). B. The *ptx-lacZ* reporter system was used to determine the activities of the BvgS_Δ65 R572L_ compared to BvgS_Δ65_ in standard conditions (0) or after growth in the presence of 2 mM chloronicotinate (CN 2). The measurements were performed at least three times, and the means and standard errors of the mean are given. Statistically different values relative to BPSM are indicated by ****, p<0.0001; ns, p>0.05. C. Quantitative RT-PCR analysis of selected genes in various recombinant strains. The values represent the Log2 values of the mean expression of each gene normalized to *bp3416* using the 2ΔCt method. The error bars represent the Ct standard deviations. BP-BvgS_Δ65_-rev79 (obtained from a mouse nose at day 14) is a hemolytic clone with BvgS_Δ65_ carrying the R^572^L substitution. BP-BvgS_Δ65_-rev26 (from nose; day 3), -rev47 (from lung; day 3), -rev54 (from nose; day 7) and -rev80 (from nose; day 14) are other hemolytic revertants without that mutation. The results represent biological triplicates for BP-BvgS_Δ65-_rev79_,_ biological duplicates for BP-BvgS_Δ65-_rev80, and single biological sampling for the other strains. In all cases, the measurements were performed in triplicates. Statistical analyses were performed using gene expression in BPSM as a control and indicated as follows: ***, p<0.001; **, p<0.01; *, p<0.05.

Finally, we characterized hemolytic colonies of BP-BvgS_Δ65_ that appeared in the first animal experiment to test the hypothesis that their *bvgS* gene harbored a mutation that locks it in the kinase mode. Such ‘constitutive’ mutants were previously reported to spontaneously occur in wt BvgS, with the substitutions mapping in particular in the PAS domain and the linker 1 [28, 29]. We thus PCR-amplified and sequenced the corresponding region of *bvgSΔ65* from a number of hemolytic clones obtained at various time points from the two sites of the mice’s respiratory tracts. A mutation replacing the CGC codon of the Arg572 residue with a Leu CTC codon was identified in a majority of the clones recovered from the lungs, and in some of those recovered from the noses (Fig 6A). Arg572 is localized in linker X of BvgS_Δ65_.

To confirm the effect of this mutation on the activity of BvgS_Δ65_, the R^572^L substitution was introduced in that variant by site-directed mutagenesis. The recombinant BvgS_Δ65-R572L_-expressing strain was hemolytic on blood agar plates irrespective of the addition of 50 mM MgSO_4_ as the modulating agent. It also showed a high level of β-galactosidase (β-gal) activity using the *ptx-lacZ* reporter fusion that did not respond to modulation (Fig 6B), unlike BP- BvgS_Δ65_. This confirms that the R^572^L mutation is sufficient to lock the BvgS_Δ65_ variant in a kinase state unresponsive to modulation.

Intriguingly, other hemolytic variants selected for sequencing did not harbor the R^572^L or any other substitution in the linker X region of BvgS_Δ65_, and whole-genome sequencing on seven such clones did not identify mutations to account for their hemolytic phenotype. We performed qRT-PCR analyses on a hemolytic variant harboring the R^572^L substitution in BvgS_Δ65_ (BP-BvgS_Δ65_-rev79) and on several other revertants devoid of that substitution. The transcription patterns of the revertant strains showed rather similar trends (Fig 6C). They expressed *cyaA* at levels similar to, or moderately lower (in BP-BvgS_Δ65_-rev80) than BPSM, in agreement with their hemolytic phenotypes on blood-agar plates. Similarly, the expression levels of *ptx* or *fimB* were similar to or slightly below those of BPSM, whereas those of another *vag*, *tcfA*, were lower. The revertants transcribed the Bvg^i^-phase gene *bipA* at levels similar to or higher than BPSM. The expression levels of the selected *vrg*, *bp2782*, were significantly lower in several of them, but not in BP-BvgS_Δ65_-rev79 harboring the R^572^L mutation, than in BPSM.

## Discussion

We genetically engineered a chimera, BvgS_Δ65_, in which the linker X of a BvgS homolog replaces the region encompassing the linker 1, the PAS domain and the linker 2 of BvgS, between the transmembrane and the DHp domains [20]. In standard growth conditions, recombinant BP-BvgS_Δ65_ is in the Bvg^i^ phase, and modulation causes an increase of BvgS_Δ65_ kinase activity, contrary to its effect on wt BvgS. We thus used the inverted regulation properties of this chimera as a tool to investigate host-pathogen interactions in an animal model of infection. Additional strains harboring other engineered versions of BvgS were also included in mouse colonization experiments. Two distinct groups of strains were thus revealed. BP-BvgS_Δ65_ and BPSM_SS1_, which are in the Bvg^i^ phase, were able to colonize and to survive in the mice noses but were rapidly cleared from the lungs, while BP-BvgS_Δ65 SS1_ could colonize and survive in those two compartments, similar to the Bvg^+^-phase control strain BPSM. There thus appears to be distinct virulence thresholds for the colonization of the mouse nose, which both Bvg^i^- and Bvg^+^-phase bacteria can colonize, and the mouse lungs, which only Bvg^+^-phase bacteria can. Our transcriptomic analyses showed that those Bvg^i^- and Bvg^+^-phase strains populate distinct ranges of phenotypes, based on gene expression levels in the various strains. Such recombinant strains are useful tools to finely dissect the interactions of *B*. *pertussis* with its host.

Our initial goal in this study was to determine whether modulation might occur in the host. Closing of the inter-lobe cavity upon ligand binding triggers signaling in other VFT domain-based systems [30, 31]. Several observations have suggested that the VFT1 domains of BvgS might function similarly. Thus, they are open in the available crystal structure, their putative solute-binding cavity is conserved among *B*. *pertussis* isolates, and their closing by inter-lobe S-S bond formation down-regulates the kinase activity of wt BvgS [12]. Therefore, we took advantage of the inverted regulation phenotype of BP-BvgS_Δ65_ to test the hypothesis that VFT1 might bind modulating ligands in the course of infection by *B*. *pertussis*. As S-S-bond-induced closing of the VFT1 domains of BvgS_Δ65_ restored kinase activity and a wt-like colonization profile by BP-BvgS _Δ65 SS1_ in a mouse model of infection (this work), ligand-induced closing of the VFT1 domains of BvgS_Δ65_ in the respiratory tract of mice might similarly have enhanced colonization by BP-BvgS_Δ65_. Our animal experiments, however, provided no indication that BP-BvgS_Δ65_ could colonize mouse lungs in the absence of reversion, arguing that modulation of BvgS activity did not occur in that organ. One caveat to our conclusion is that temporally or spatially restricted *in vivo* modulation might be missed in animal experiments. Of note, high-level expression of several *vrgs* in mouse infection were recently reported [32]. Using a fluorescent reporter under the control of the *ptx* promoter in BP-BvgS_Δ65_, we also found no evidence that modulation might occur inside macrophages or dendritic cells (our unpublished data), in line with recent proteomic analyses of *B*. *pertussis* that showed increased production of some virulence proteins inside human macrophages [33]. Nevertheless, as both Bvg^i^- and Bvg^+^-phase strains can colonize the nose, we cannot exclude the possibility of up-modulation of BvgS_Δ65_ in the upper respiratory tract of mice. It is also possible that modulating signals are present in specific environments or conditions encountered by the bacteria in the human respiratory tract, but not in that of the mouse.

In the first animal experiment, colonies with hemolytic phenotypes progressively outnumbered non-hemolytic colonies. Transcriptomic analyses of those variants showed increased expression levels of several virulence factors relative to parental BP-BvgS _Δ65_. The rapid increase of the proportions of those more virulent revertants and their ability to outcompete non-hemolytic clones suggest that they were selected for by innate immune responses in the mice, and that this selective pressure is very strong in the lungs. In the nose, in contrast, non-hemolytic bacteria in the Bvg^i^ phase could survive for up to four weeks, similarly to those in the Bvg^+^ phase. Nevertheless, the occurrence of both BP-BvgS_Δ65_ and hemolytic revertants in the noses of mice from day 3 *de facto* resulted in a mixed infection, and the initially low proportions of revertants in the noses increased relative to their Bvg^i^-phase parent over time. This shows that they may have a selective advantage in the upper respiratory tract as well, in agreement with previous reports that the prototypical Bvg^i^-phase-locked strain harboring the T^733^M substitution, was less competitive in both compartments [26]. Nonetheless, as selective pressure is less intense in the nose, it is conceivable that attenuated mutants could persist in that compartment in the absence of more virulent competitors. The report that avirulent *B*. *pertussis* clones harboring an IS481 insertion in *bvgAS* were detected in the nasopharynx of experimentally infected monkeys suggests that even avirulent bacteria might be able to persist for some time in the upper respiratory tract once infection has been established [34].

The transcription profile of BP-BvgS_Δ65-SS1_ shows that it is not a fully virulent Bvg^+^-phase strain, but an additional intermediate between the *bona fide* Bvg^i^ and Bvg^+^ phenotypes. Nevertheless, expression of *vags* in that strain appears to be sufficient to establish lung infection, indicating that the virulence phenotype of BP-BvgS_Δ65-SS1_ is beyond the threshold at which mouse lung colonization can occur. Whether such a moderately virulent strain might be successful in natural human infections is unknown, and it might be interesting to address this question in a baboon model of infection [1]. Its phenotype might be advantageous in the face of the immune pressure, notably linked to vaccination. The loss of specific virulence factors or lower levels of virulence gene expression have indeed been reported to occur in currently circulating *B*. *pertussis* strains [35–39]. On the contrary, however, immune pressure appears to currently select for strains that express greater levels of pertussis toxin [40]. Thus, distinct virulence factors make different contributions to the success of infection. In this respect, it is worth noting that a live attenuated vaccine candidate, BPZE1, which lacks some *bona fide* virulence factors, efficiently colonizes the respiratory tract of animals and humans without causing disease [41, 42].

*B*. *pertussis* has conserved its capability to shift between the Bvg^+^ phase and the Bvg^-^ or Bvg^i^ phases in the laboratory. However, the relevance of the latter phases in the host and the conditions that trigger phenotypic modulation in its current lifestyle remain unclear. For *B*. *pertussis*, the shift to the Bvg^-^ phase might be a remnant of an ancestor able to survive outside mammalian hosts, as the Bvg^-^ phase of *Bordetella bronchiseptica* allows its growth and dissemination in amoeba as an environmental reservoir [43]. The occurrence and the role of the Bvg^i^ phase in the infection remain unclear as well. The presence of antibodies directed against Bvg^i^-phase-specific antigens in the sera of convalescent children was reported [44] but contradicted by subsequent work [26]. An *in vivo* shift to the Bvg^i^ phase in the nasopharynx might be favored by the somewhat lower temperature there than in the rest of the respiratory tract, but a recent study revealed that virulence factor expression in *B*. *pertussis* persists at suboptimal temperatures [45]. Several studies including this one have shown that Bvg^i^-phase locked *B*. *pertussis* strains are proficient for infection of the mouse nose. However, Bvg^+^-phase locked *B*. *bronchiseptica* remains proficient for infection and transmission in a pig model of disease, casting doubt on the idea that the Bvg^i^ phase contributes to transmission [27]. It is possible that the bacteria transiently shift to the Bvg^i^ phase before or after transmission, but the Bvg^i^ phase passes undetected by the immune system.

It was proposed that virulence genes expressed by *B*. *pertussis* could be classified in four classes, and that *vrgs* were expressed mainly in absence of phosphorylated BvgA, that is, in the Bvg^-^ phase [4]. Nevertheless, we showed in this study that expression of several *vrgs* was at similarly high levels in the Bvg^i^-phase strains as in the modulated, Bvg^—^phase wt strain. In contrast, *vrgs* were not overexpressed in the Bvg^i^-phase-locked strain described earlier [23]. Whether these differences stem from the different genetic backgrounds of the strains used in the two studies remains to be determined. It is also possible that the *vrgs* form distinct subgroups that are regulated in slightly different ways, as shown for *vags* [24].

The majority of hemolytic revertants of BPSM-BvgS_Δ65_ isolated from the mice had a specific mutation in linker X that results in the substitution of an Arg residue with a Leu. In the subset of BvgS homologs devoid of a PAS domain, the linker X harbors two antagonistic coiled-coil registers, and regulation of activity implies interconversion between the two marginally stable coiled coils defined by those registers [20]. In BvgS_Δ65_, Arg572 appears to be involved in repulsive interactions between the two helices in the kinase register of the coiled coil, but not in the phosphatase register [20]. Therefore, the removal of unfavorable interactions by the Arg to Leu replacement is likely to stabilize the two-helix coiled coil in the kinase register, which might lock BvgS_Δ65_ in the kinase state of activity.

Finally, some of the hemolytic revertants from BPSM-BvgS_Δ65_ isolated from the noses of mice at early time points, did not harbor the R^572^L point mutation to account for their hemolytic phenotype. While whole-genome sequencing did not enable us to identify any other specific mutation, restoration of a hemolytic phenotype may have arisen from genomic reshuffling of insertion sequences or epigenetic modifications. The observation that the hemolytic phenotype appears to be maintained upon sub-culturing suggests that the BvgS_Δ65_ variant might generate bistability, in particular in the mouse nose. Of note, bistable phenotypes have been reported in the lower respiratory tract of mice infected with specific mutants of the broad-host range pathogen *Bordetella bronchiseptica* [46].

## Supporting information

S1 Table Raw data of the RNA seq experiments. The RPKM for each open reading frame are provided for the 6 conditions. The corrected p-values and the fold changes (FC) are also provided. Fold changes > 2 or >-2 (in log2) are considered to be significant.
(XLSX)
